# Prevalence of Common Diabetes Mellitus Misinformation Exposure, Cognitive Attitude, and Intention to Share Information Among Patients in a Primary Care Unit

**DOI:** 10.3390/healthcare13141762

**Published:** 2025-07-21

**Authors:** Thanapol Pratueangpong, Napakkawat Buathong, Phoomjai Sornsenee

**Affiliations:** Department of Family Medicine and Preventive Medicine, Faculty of Medicine, Prince of Songkla University, Hat Yai District, Songkhla 90110, Thailand; prthanapolth@gmail.com (T.P.); napakkawatb@gmail.com (N.B.)

**Keywords:** diabetes mellitus, misinformation, cognitive attitude, information sharing

## Abstract

**Background/Objectives:** Misinformation significantly impacts self-care behaviors and treatment outcomes in patients with type 2 diabetes mellitus (T2DM). We investigated the prevalence and content of diabetes-related misinformation among Thai patients with T2DM, examining the influence on cognitive attitudes and intentions to share such information. **Methods**: We employed a mixed-methods approach, conducting initial qualitative interviews with healthcare professionals and patients with T2DM to identify key misinformation themes. These themes guided the development of a validated questionnaire that was distributed to 107 patients with T2DM. Spearman’s correlation and multiple linear regression analyses were used to assess the relationships between misinformation exposure, attitudes, and sharing intentions. **Results**: Misinformation was categorized into four domains: medication side effects, alternative treatments, imbalanced lifestyle, and symptom perception. Exposure to misinformation ranged from 19.6% to 94.4%, with word of mouth identified as the primary source (81.18%). Misconceptions regarding symptom perception and alternative treatments were most prevalent. Information source, especially healthcare providers (β = 0.4); personal attitudes towards misinformation (β = 0.24); and exposure level (β = 0.46) significantly influenced the intention to share misinformation. **Conclusions**: This study highlights the need for targeted educational interventions to address widespread misconceptions in the management of T2DM, particularly those related to symptom perception and alternative treatments. Addressing these misinformation sources may be associated with improved self-management practices and could inform strategies aimed at enhancing patient outcomes.

## 1. Introduction

Type 2 diabetes mellitus (T2DM) remains a critical global health challenge, impacting over 422 million individuals worldwide. Furthermore, complications relating to T2DM account for 1.5 million deaths annually. With the increasing incidence of diabetes, the Global Diabetes Compact of the World Health Organization seeks to unite stakeholders in reducing the risk of diabetes and ensuring equitable access to comprehensive and affordable care [1]. This highlights the critical need for effective management strategies that integrate professional healthcare guidance with robust patient self-care practices [2,3].

Misinformation (i.e., inaccurate information about their care) poses a significant challenge for patients with diabetes. Such misinformation includes erroneous beliefs about dietary management, medication effects, and alternative treatments. When patients internalize such misinformation, it can potentially form misconceptions that severely undermine self-care and adherence to treatment protocols [4,5]. Crucially, misinformation actively promotes the propagation of falsehoods, which differ from a simple lack of knowledge. In this study, the term “misinformation” refers specifically to inaccurate or false content that is shared with or communicated to patients, while “misconceptions” denote the resulting incorrect beliefs held by patients, and “lack of knowledge” refers to gaps where patients have not received any information. Exacerbated by the rapid advancement of digital technologies, such misinformation is often disseminated widely via social media and various online platforms [6]. The consequences of the resulting misconceptions are profound, including poor glycemic control and increased complications, thereby compromising the efficacy of diabetes management and adversely impacting patient outcomes [7,8,9].

In Thailand, the prevalence of diabetes among adults has slightly increased, with recent data indicating that a substantial proportion of the population with diabetes remains poorly controlled, leading to increased mortality, reduced quality of life, and elevated healthcare costs [10,11]. To address these issues, comprehensive strategies are being implemented; however, studies focusing on diabetes self-care misinformation remain limited within the country [12,13].

The findings from this study are poised to enhance patient education and engagement, thus bolstering effective diabetes management strategies. This will enable an in-depth analysis of the connections between exposure to misinformation, cognitive attitudes toward it, and its dissemination among patient communities [14,15,16]. In this study, we aimed to explore the prevalence of misinformation among Thai patients with diabetes and understand how it shapes their perceptions and behaviors. Using a mixed-methods approach, the initial phase involved qualitative interviews with healthcare providers and patients to identify prevalent misinformation themes within the diabetes community. These findings will inform the creation of a structured questionnaire to quantitatively measure the extent and impact of misinformation. In addition to describing the prevalence and content of misinformation, this study aimed to explore whether exposure to misinformation was associated with more favorable cognitive attitudes and whether such attitudes could be related to the intention to share misinformation. These analyses were intended to provide preliminary insights into these potential relationships to inform the development of targeted interventions.

## 2. Materials and Methods

### 2.1. Ethics Approval

This study was approved by the Human Research Ethics Committee, Faculty of Medicine, Prince of Songkla University, on 9 May 2023 (approval number: REC 66-168-9-4). This study was conducted in accordance with the Declaration of Helsinki.

Informed consent was obtained from all participants before data collection for both the interview and questionnaire groups. Data confidentiality and secure storage were ensured, with voice recordings, transcribed text, and all questionnaires being fully computerized, encrypted with passwords, and anonymized by the researchers to maintain confidentiality.

### 2.2. Study Design

This study employed a two-phase mixed-methods design conducted in a primary care unit at Songklanagarind Hospital in Southern Thailand between April 2023 and May 2024. The first phase involved conducting qualitative interviews to gather insights into the prevalence of misinformation among patients with T2DM and their care providers. The insights obtained were used to develop a structured questionnaire for the second phase.

### 2.3. Participants and Data Collection

#### 2.3.1. Phase I

This study included physicians and nurses with at least 5 years of experience in diabetes care, in addition to nutritionists specializing in diabetes management. Patients with T2DM aged between 18 and 60 years were also included. Patients with T2DM evaluated as having frailty by one of the researchers were excluded. Interviews lasted 20–30 min and included a research explanation and consent process, which were conducted at the hospital. Data were collected through voice recording, transcribed into text, and then stored on encrypted computers accessible only to the authorized research team. The guide question interview form was developed based on a literature review, encompassing domains such as diabetes medication, diet, exercise, and herbal use [8,9,17,18,19]. Researchers determined that data saturation was achieved after conducting 14 interviews, including two physicians, two nurses, two nutritionists specializing in diabetes care, and eight patients with T2DM. As similar themes consistently emerged across participants, particularly regarding medication side effects, alternative treatments, dietary habits, exercise patterns, and symptom perceptions, additional interviews were deemed unlikely to yield substantially new insights.

#### 2.3.2. Phase II

This phase included patients with T2DM diagnosed according to ICD-10 for at least 1 year, who had undergone at least three follow-up visits at the primary care unit and could communicate in Thai. We excluded patients deemed unable to provide reliable answers by the research team. Participants were recruited through invitation posters at the hospital. After the informed consent process, they completed a paper-based questionnaire, with data collection being anonymous.

The questionnaire was developed from the thematic analysis in Phase I and included sections on demographic data, misinformation, cognitive attitude, and intention to share. Content validity was established through an Item-Objective Congruence (IOC) index assessed by three independent experts in diabetes care and health communication. All items demonstrated IOC values > 0.5 and were considered acceptable. The questionnaire was also piloted among a small group of 32 patients before full deployment to assess clarity and comprehension. Its internal consistency reliability was confirmed, achieving a Cronbach’s alpha of 0.79. Specific details on the type of misinformation addressed in the questionnaire are shown in Table 1.

### 2.4. Statistical Analysis

Qualitative data from Phase I were analyzed using thematic analysis to identify meaningful patterns and themes related to misinformation. Data collection was performed using Google Forms^®^ (Google LLC, Mountain View, CA, USA), and the collected data were processed using Microsoft Excel^®^ (Microsoft Corporation, Redmond, WA, USA). Detailed statistical analyses were conducted using R version 4.2.3 (R Foundation for Statistical Computing, Vienna, Austria). The questionnaire included sections on demographic data, misinformation exposure, cognitive attitudes, and intention to share misinformation. The cognitive attitude and intention scales comprised multiple items rated on a 5-point Likert scale (1 = strongly disagree to 5 = strongly agree). Composite scores were calculated by summing item responses, with higher scores indicating more favorable attitudes or stronger intentions. For the quantitative analysis, demographic variables, such as age, sex, socioeconomic status, and religion, were examined to provide an overview of participant characteristics. These variables were assessed alongside clinical characteristics, including the duration after diabetes diagnosis, lifestyle modifications without medication, use of oral anti-diabetic drugs, insulin use, and diabetes complications.

The assessment of these variables utilized frequencies, percentages, means, and standard deviations to quantify and describe the sample.

Following the initial descriptive analysis, univariate analyses were conducted to identify potential influences on cognitive attitudes towards misinformation and intentions to share it. A significance level of *p* < 0.20 was used to select variables for the multivariable analysis. This process further examined the relationships between demographic and clinical characteristics and both cognitive attitudes and intentions to share misinformation. The variables for cognitive attitude and intention to share were transformed from nominal to continuous scales to meet the analytical requirements. To refine the model and determine the most significant predictors of misinformation sharing behaviors, a backward stepwise regression was employed using the Akaike information criterion. Model diagnostics included assessments of residual normality (Shapiro–Wilk test and Q–Q plots), homoscedasticity (Breusch–Pagan test and residuals vs. fitted plots), multicollinearity (variance inflation factor), and examination of standardized residuals. Post hoc power analyses were conducted. Effect sizes were calculated as Cohen’s f^2^ derived from the adjusted R^2^ of each model. The statistical power was estimated based on the number of predictors and degrees of freedom.

## 3. Results

### 3.1. Baseline Characteristics

A total of 107 patients with T2DM completed the questionnaire between December 2023 and February 2024. The mean age of participants was 64.5 ± 9.8 years. Most participants were female (59.8%), Buddhist (94.4%), had primary education (45.8%), and reported a monthly income of < THB 10,000 (58.9%). The median duration since diabetes diagnosis was 7 years (IQR 3.5–10), with the majority receiving oral anti-diabetic drugs (84.1%). Dyslipidemia was the most common comorbidity (93.5%), and only 2.8% had developed complications related to diabetes (Table 2).

### 3.2. Prevalence of Exposure and Intention to Share Misinformation on T2DM

**Figure 1:**
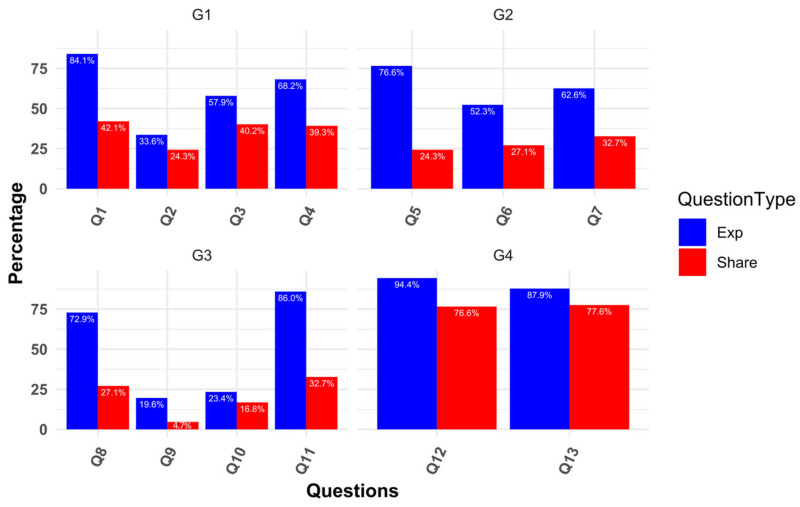
Percentage of misinformation exposure and intention to share for each question. G1 = side effects of medication; G2 = alternative treatment; G3 = imbalanced lifestyle; G4 = symptom perception; Exp = Exposure.

The prevalence of exposure to misinformation among patients with T2DM was highly variable, ranging from 19.6% to 94.4% (Figure 1). Regarding the intention to share such misinformation, 77.6% of participants were likely to share the belief that patients without symptoms manage their diabetes well, and 76.6% endorsed the notion that sugary drinks are beneficial during fatigue. In contrast, the misinformation statement “patients with diabetes on insulin cannot exercise” was the least shared, with only 4.7% of participants endorsing it.

**Figure 2:**
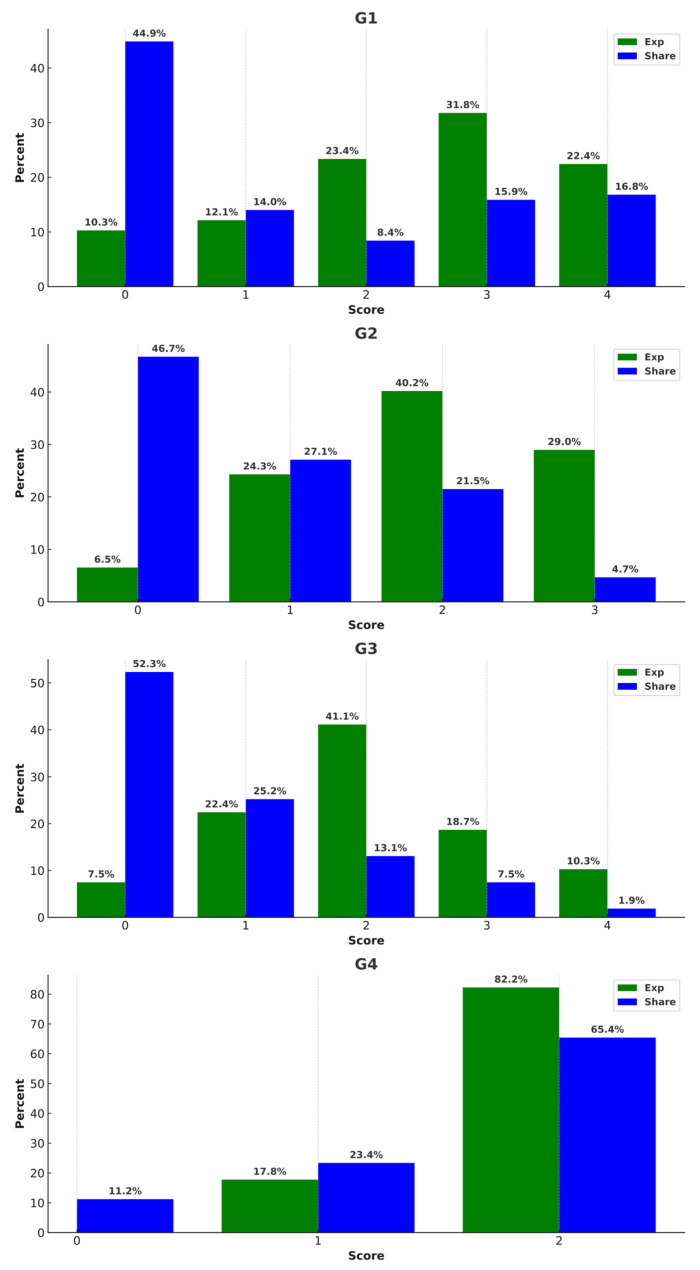
Percentage of misinformation exposure and intention to share in each group. G1 = side effects of medication; G2 = alternative treatment; G3 = imbalanced lifestyle; G4 = symptom perception; Exp = exposure.

A detailed examination of the prevalence and intention to share misinformation across various categories is presented in Figure 2. For example, in Group 1 (G1), which includes four items on medication side effects, 31.8% of participants had an exposure score of 3—meaning they encountered misinformation in three out of four items. Similarly, in G4 (symptom perception), 82.2% of participants scored the full 2 points, indicating they were exposed to both misinformation items in this group. Notably, intention to share was assessed at the time of reading, independent of prior exposure—capturing participants’ immediate willingness to share each statement.

In G2 (alternative treatments), despite moderate exposure levels, 46.7% of participants had a sharing score of 0, indicating hesitation to spread such content. In contrast, G4 showed both high exposure and high intention to share, with 65.4% of participants intending to share both symptom-related items. These patterns suggest that participants were more cautious about sharing treatment-related misinformation than symptom-based misconceptions.

### 3.3. Sources of Misinformation

Individuals without diabetes were identified as the primary source of misinformation, influencing 46.21% of participants (Table 3). In addition, both patients with diabetes and healthcare providers contributed to the spread of misinformation.

### 3.4. Cognitive Attitudes Towards Misinformation

**Figure 3:**
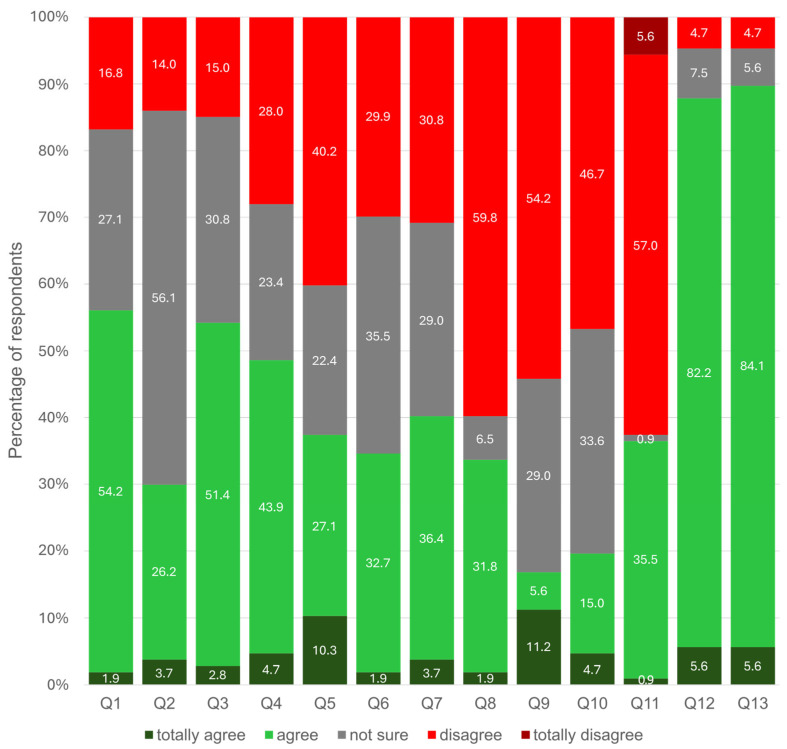
Distribution of cognitive attitudes towards diabetes misinformation (n = 107).

Figure 3 details the cognitive attitudes towards misinformation among the participants. A total of 89.7% of participants agreed with the misinformation statement that patients with diabetes mellitus without any symptoms are those who control their blood sugar levels well (Q13), and 87.8% incorrectly believed that sugary drinks can alleviate symptoms of fatigue (Q12), both of which fall under the symptom perception group (G4), indicating that most participants accepted these misconceptions.

Conversely, most participants rejected the misinformation statements in G3, the imbalanced lifestyle group (Q8–Q11). Disagreement was highest in Q8 (59.8%) and Q11 (62.6% when combining “disagree” and “totally disagree”), suggesting stronger rejection of lifestyle-related misconceptions. Responses in G1 (medication side effects) and G2 (alternative treatments) were more varied, with no clearly dominant patterns. This distribution illustrates that while some forms of misinformation are widely accepted, others are subject to more critical appraisal, reflecting a varied understanding of diabetes management among the sample.

### 3.5. Correlation Between Exposure, Attitude, and Intention to Share

**Figure 4:**
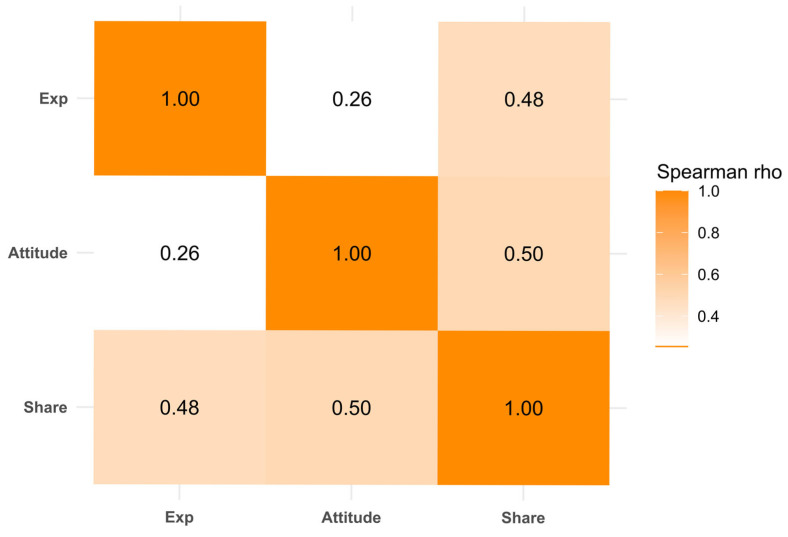
Correlation heatmap of exposure, cognitive attitude, and intention to share. Exp = exposure; Attitude = cognitive attitude; Share = intention to share.

Figure 4 presents a correlation heatmap illustrating the relationships among exposure to misinformation, cognitive attitudes, and the intention to share. The intention to share showed a moderate positive correlation with cognitive attitudes (Spearman’s rho = 0.50) and a slightly lower correlation with exposure (rho = 0.48). The weakest association was between exposure and cognitive attitudes (rho = 0.26). These findings suggest that while exposure is relevant, cognitive attitudes may play a more central role in shaping patients’ willingness to share misinformation.

**Figure 5:**
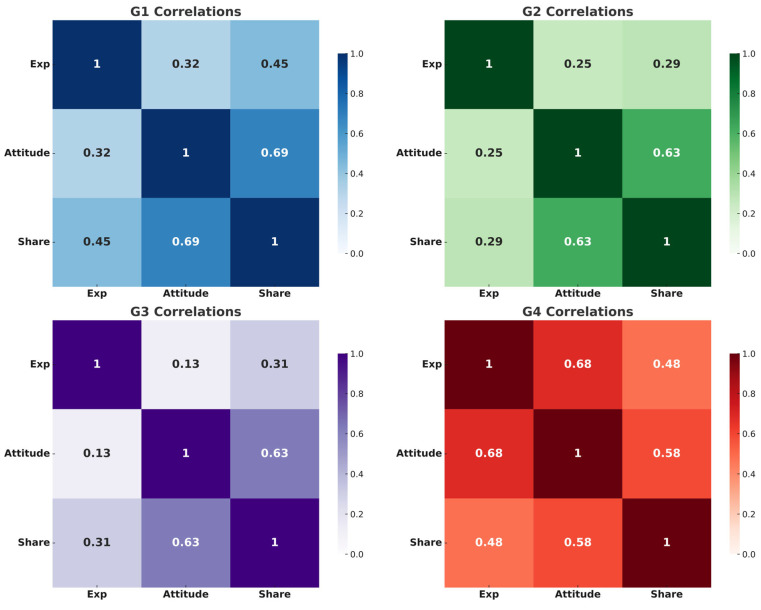
Correlation heatmaps of exposure, cognitive attitude, and intention to share in each misinformation group. G1 = side effects of medication; G2 = alternative treatment; G3 = imbalanced lifestyle; G4 = symptom perception; Exp = exposure; Attitude = cognitive attitude; Share = intention to share.

In Figure 5, the analysis explores the correlations within each group of misinformation. This detailed view reveals how the dynamics of misinformation vary significantly across different categories. For example, in the side effects of medication group (Group 1), a tendency existed for those exposed to this type of misinformation to share it, influenced heavily by their cognitive attitudes. Conversely, in the symptom perception group (Group 4), the data show a robust correlation between exposure and both attitude and intention to share, suggesting that exposure strongly shapes how participants perceive and decide to disseminate misinformation. Each group displays unique correlation patterns, underscoring the need for tailored approaches to address misinformation based on the specific misconceptions prevalent in each category.

### 3.6. Statistical Analysis of Factors Influencing Misinformation Among Patients with T2DM

The univariate analysis revealed significant associations between specific demographic and clinical characteristics and the intention to share misinformation, as well as cognitive attitudes, among patients with T2DM (Table 4). Sex, education level, and average monthly income had a significant influence on the intention to share misinformation (*p* < 0.05 for all). Specifically, females, individuals without formal education, and those with lower income levels were more likely to share misinformation.

In Table 5, the source of information from healthcare providers is shown to have a positive influence on cognitive attitudes towards diabetes-related misinformation (β = 0.97, 95% confidence interval (CI) = 0.43–1.51, *p* < 0.01). Conversely, higher education levels did not significantly impact cognitive attitudes, with primary education showing a non-significant beta coefficient of −1.72 (95% CI (8.42, 4.99), *p* = 0.61) and education above primary level showing −4.84 (95% CI (−11.51, 1.83), *p* = 0.15).

For the intention to share misinformation, the presence of comorbidities, such as atrial fibrillation, benign prostate hyperplasia, or gout, showed a negative association (β = −2.04, 95% CI (−3.88, −0.20), *p* = 0.03). Furthermore, patients on oral anti-diabetic drugs were more likely to share misinformation (β = 2.01, 95% CI (0.50, 3.52), *p* = 0.01), while those on insulin therapy showed no significant difference (β = −0.28, 95% CI (−2.56, 1.99), *p* = 0.80). The regression models also demonstrated significant positive relationships between the intention to share misinformation and factors such as exposure to misinformation (β = 0.46, 95% CI (0.29, 0.63), *p* < 0.01) and cognitive attitude scores (β = 0.24, 95% CI (0.15, 0.33), *p* < 0.01), highlighting the critical influence of these variables.

In the multiple linear regression models, the Shapiro–Wilk test indicated minor non-normality in the shared model residuals (W = 0.9659, *p* = 0.0076), while the attitude model showed no significant deviation (W = 0.9838, *p* = 0.219). The Breusch–Pagan test suggested heteroscedasticity in the shared model (*p* = 0.045) but not in the attitude model (*p* = 0.756). Variance inflation factor values were low (all <1.3), indicating no notable multicollinearity. The shared model demonstrated an adjusted R^2^ of 0.50 with a residual standard error of 2.20, while the attitude model had an adjusted R^2^ of 0.20 with a residual standard error of 4.64. Robust standard errors were applied in the shared model to improve inference reliability. Overall, the results should be interpreted with caution due to these minor assumption violations and the exploratory nature of the analysis. In the model predicting the intention to share misinformation, the adjusted R^2^ was 0.50, corresponding to a large effect size (Cohen’s f^2^ = 1.0) and an achieved power of 1.0 (100%). For the model predicting cognitive attitudes, the adjusted R^2^ was 0.20, corresponding to a medium-to-large effect size (Cohen’s f^2^ = 0.25) and an achieved power of 0.991 (99.1%).

## 4. Discussion

The primary objective of this study was to examine the prevalence and impact of misinformation among patients with T2DM and to identify factors that influence the dissemination of such misinformation. Our findings reveal a significant exposure to misinformation among the patient population, particularly regarding medication, alternative treatments, lifestyle, and disease symptom management. Most patients with diabetes encountered misinformation through word-of-mouth rather than online sources. This pattern may be associated with the demographic characteristics of the participants, such as older age and lower income, which may limit their access to and interaction with online platforms. Furthermore, these demographics may also explain why patients generally displayed moderate attitudes toward sharing misinformation. Misinformation transmitted via word-of-mouth among both individuals with and without diabetes can often be distorted, depending on the intentions of the speakers, contributing further to the challenge of managing diabetes effectively [20,21].

One notable example of a misconception within the symptom perception category, which is the most frequently encountered and shared type of misinformation, was the management of fatigue. Patients often mistook signs of fatigue for hypoglycemia, which may have led them to consume hyperglycemic foods or drinks unnecessarily, under the assumption that they were experiencing a drop in blood sugar. This misconception highlights the complexities involved in diabetes symptoms and suggests potential risks associated with self-directed treatments based on inaccurate information. Fatigue in patients with diabetes is a multifaceted issue, involving both physiological and psychological factors [22]. The erroneous assumption that hypoglycemia is the only cause of fatigue and requires immediate glucose intake carries the risk of exacerbating the condition and contributing to harmful self-treatment practices [23].

Misinformation about dietary choices for patients with diabetes was highly prevalent, echoing findings from prior research that categorized foods as “good” or “bad.” Often, “good” foods are perceived as consumable without limits, and “bad” foods are viewed as entirely avoidable [24]. Notably, some of this misinformation originates from healthcare providers, possibly due to communication challenges during diabetes care appointments [25]. These challenges may be related to an excessive focus on glycemic control or concerns about hypoglycemia, which may contribute to generalized dietary advice that does not tailor to individual patient needs. The trust patients place in their healthcare providers significantly affects the spread of misinformation. When patients receive and trust flawed advice due to their strong belief in these professional sources, they are more likely to accept and propagate this information among their peers [26].

The social learning theory posits that individuals acquire behaviors, attitudes, and beliefs through observation, imitation, and reinforcement from social interactions and experiences [27]. Patients are particularly inclined to share misinformation that they have personally endorsed or encountered previously. This tendency is exacerbated by repeated exposure, which increases the likelihood of its dissemination—a concept supported by studies on social reinforcement [28]. The social identity theory suggests that patients with diabetes often trust and value information shared by peers undergoing similar experiences and challenges, reinforcing the cycle of misinformation [29].

Notably, patients treated with oral anti-diabetic drugs were found to often share misinformation, possibly due to prevalent misconceptions regarding these treatments. This aligns with findings from a previous study, which showed that these patients, although generally knowledgeable about their condition, may harbor fears about treatment changes, which could contribute to the dissemination of misinformation among peer groups [30]. Considering our findings regarding insulin—which represents a more advanced stage of treatment—patients on oral medications might view transitioning to insulin injections as an escalation in disease severity. This perception might contribute to reluctance and the sharing of misinformation about insulin treatments. This observation underscores the importance of further research to fully understand these dynamics and develop targeted educational strategies that address specific misconceptions. Our findings highlight significant gaps in the knowledge and widespread acceptance of diabetes-related misinformation, reflecting global trends noted in similar studies [7,8,9,17,18,19,23,24,30]. These misconceptions often originate from traditional beliefs and modern digital misinformation channels. Addressing these misconceptions through tailored educational programs could significantly enhance patient engagement and adherence to scientifically validated diabetes management practices.

In addition to traditional educational approaches, it is essential to consider how modern digital environments contribute to the dissemination of misinformation, which presents opportunities for targeted interventions. In the context of increasingly networked digital environments, social media platforms serve not only as sources of exposure but also as conduits for peer endorsement and rapid dissemination of health misinformation [31,32,33,34]. Addressing this dynamic requires interventions that extend beyond simply correcting false information; it should instead leverage the same platforms to promote credible content and counteract misperceptions. For example, community-based digital campaigns that incorporate trusted messengers and interactive educational materials have demonstrated promise in reducing susceptibility to misinformation and curbing its spread [31,33]. In addition, interventions that provide patients with decision aids and credible resources at the point of care may help mitigate the influence of online peer networks [32,34]. Given that misinformation can be reinforced through social validation within digital communities, strategies that foster critical evaluation skills and equip patients to question the credibility of information encountered online are essential [33,34]. These considerations align with frameworks, such as the cognitive dissonance theory, which proposes that individuals may reject information conflicting with their existing beliefs to reduce psychological discomfort [35]. Additionally, variations in health literacy and digital health literacy likely influence patients’ ability to critically appraise information and differentiate credible sources from misinformation, highlighting the importance of integrating these concepts into future intervention strategies [36].

### 4.1. Strengths and Limitations

One of the major strengths of this study is its mixed-methods approach, which provides a comprehensive understanding of the types and sources of misinformation and their consequences on patient behavior. The use of both qualitative interviews and quantitative assessments enables a robust analysis of the data, providing detailed insights into patient perceptions and actions.

However, this study has some limitations. The sample size, though adequate for a preliminary analysis, may not fully represent the broader T2DM population in Thailand or other regions. In addition, while content validity and internal consistency were evaluated, further psychometric development of the measurement tools, including exploratory and confirmatory factor analyses (EFA/CFA), would be necessary to confirm their construct validity and enhance their overall reliability. Recruitment through invitation posters at a single hospital in Southern Thailand may have introduced selection bias toward more health-conscious participants. The relatively homogeneous sample, in terms of age, socioeconomic status, and cultural background, further limits generalizability. In addition, the reliance on self-reported data could have led to inaccuracies in reporting behaviors, particularly cognitive attitudes and intentions to share misinformation. Although post hoc power analyses indicated adequate power to detect medium-to-large effect sizes in both models, the absence of an a priori sample size calculation limits the interpretability of non-significant results and may have reduced sensitivity to smaller associations. Finally, the modest sample size and minor violations of regression assumptions warrant cautious interpretation of these exploratory findings. As multiple statistical tests were conducted without formal correction for multiplicity, there is an increased risk of Type I errors.

### 4.2. Future Research Suggestions

Future research should focus on larger and more diverse populations to validate these findings and assess the effectiveness of different educational interventions. Interventional studies that tailor educational content based on specific misconceptions identified through similar mixed-methods approaches could be particularly effective. Additionally, exploring the impact of digital misinformation, especially from social media, would provide valuable insights given the rising influence of these platforms on health behaviors. Future studies should also incorporate clinical outcome measures, such as HbA1c levels, to evaluate the direct impact of misinformation on disease control. Finally, further psychometric development of measurement tools, including EFA/CFA, would strengthen the reliability and validity of instruments assessing misinformation exposure and attitudes.

## 5. Conclusions

This study highlights the significant impact of misinformation on diabetes management and the critical role of healthcare providers in addressing these misconceptions. Prioritizing educational strategies and enhancing provider–patient communication may support improved treatment engagement and help address widespread misconceptions in diabetes management. Future strategies should incorporate these elements, tailored to address specific misconceptions and delivered through platforms that patients frequently access, to maximize reach and potentially support more effective management of diabetes-related misinformation.

## Figures and Tables

**Table 1:** Grouping of misinformation topics.

Group	Misinformation Question
Side effects of medication (Group 1)	1. Long-term use of diabetes medication leads to kidney impairment.
	2. Long-term use of insulin injections leads to kidney impairment.
	3. Using multiple types of diabetes medication together accelerates kidney impairment.
	4. If a doctor starts diabetes treatment with insulin injections, patients must keep injecting for life.
Alternative treatment (Group 2)	5. Using herbs to treat diabetes might make the disease go away completely.
	6. Using sugar substitutes can lower blood sugar levels.
	7. Patients with diabetes mellitus can take supplements instead of controlling their diet.
Imbalanced lifestyle (Group 3)	8. Patients with diabetes mellitus do not need to limit their food intake if the food is not sweet.
	9. Patients with diabetes mellitus who inject insulin are not able to exercise.
	10. Patients with diabetes mellitus who have experienced hypoglycemia during exercise may not be able to exercise again.
	11. Patients with diabetes mellitus cannot eat fruits that are sweet in taste at all.
Symptom perception (Group 4)	12. When patients with diabetes mellitus experience fatigue, it is necessary to drink sugary beverages to alleviate the symptoms quickly.
	13. Patients with diabetes mellitus without any symptoms are those who control their blood sugar levels well.

**Table 2:** Participants’ baseline characteristics (n = 107).

Sociodemographic Characteristics	Patients (n = 107)
Age (years), mean (SD)	64.5 (9.8)
Sex, n (%)	
Male	43 (40.2)
Female	64 (59.8)
Religion, n (%)	
Buddhism	101 (94.4)
Islam	6 (5.6)
Education level, n (%)	
No formal education	2 (1.9)
Primary education	49 (45.8)
Secondary education	18 (16.8)
Vocational education/professional bachelor’s degree	13 (12.1)
Bachelor’s degree	19 (17.8)
Above a bachelor’s degree	6 (5.6)
Average monthly income (THB), n (%)	
≤10,000	63 (58.9)
10,001–20,000	22 (20.6)
>20,000	22 (20.6)
Time since diagnosis (years), median (IQR)	7 (3.5–10)
Dyslipidemia, n (%)	100 (93.5)
Hypertension, n (%)	72 (67.3)
Another comorbidity, n (%)	6 (5.6)
Current diabetes treatment, n (%)	
Insulin therapy	11 (10.3)
Oral anti-diabetic drug	90 (84.1)
Lifestyle modifications (no medication)	6 (5.6)
Current diabetic retinopathy	3 (2.8)

**Table 3:** Sources of misinformation among study participants *.

Source	Times of Exposure, n (%) *
Non-person	
Television and radio	78 (7.12)
Online media (Internet, YouTube, Facebook, Twitter)	128 (11.69)
Person	
Patients with diabetes mellitus	244 (22.28)
Patients without diabetes mellitus	506 (46.21)
Healthcare providers	139 (12.69)

* Total possible exposure times = 6955 times (107 participants × 5 sources × 13 questions).

**Table 4:** Univariate factors associated with cognitive attitude and the intention to share misinformation on T2DM.

Factors		Cognitive Attitude			Intention to Share		
Sociodemographic Characteristics	Patients (n = 107)	Score	SD	*p*-Value	Score	SD	*p*-Value
Age, years				0.55			0.58
≥65	49	3.152	0.967		0.350	0.477	
<65	58	3.199	0.913		0.365	0.481	
Sex				0.03 *			0.01 *
Male	43	3.077	0.944		0.315	0.464	
Female	64	3.245	0.929		0.431	0.495	
Religion				0.30			0.74
Buddhism	101	3.187	0.936		0.357	0.479	
Islam	6	3.013	0.967		0.372	0.493	
Educational level				<0.001 *			<0.001 *
No formal education	2	3.308	0.928		0.615	0.487	
Primary education	49	3.306	0.911		0.394	0.489	
Above primary education	56	3.060	0.949		0.317	0.465	
Average monthly income, THB				0.02 *			<0.001 *
≤10,000	63	3.250	0.920		0.410	0.492	
>10,000	44	3.073	0.955		0.283	0.451	
Time since diagnosis, years				0.82			0.61
<10	62	3.170	0.948		0.352	0.478	
≥10	45	3.188	0.926		0.366	0.482	
Dyslipidemia				0.34			0.32
No	7	3.319	0.863		0.407	0.491	
Yes	100	3.168	0.943		0.355	0.478	
Hypertension				0.16			0.12
No	35	3.099	0.924		0.330	0.470	
Yes	72	3.216	0.943		0.372	0.483	
Another comorbidity				0.57			0.03 *
No	101	3.172	0.942		0.365	0.481	
Yes	6	3.269	0.872		0.244	0.429	
Current diabetes treatment				0.08			0.15
Insulin therapy	11	3.469	1.070		0.378	0.485	
Oral anti-diabetic drug	90	3.138	0.923		0.362	0.481	
Lifestyle modification (no medication)	6	3.231	0.799		0.256	0.439	
Diabetic complications				0.50			0.09
Current diabetic retinopathy	3	3.173	0.942		0.487	0.506	
No diabetic complications	104	3.333	0.795		0.354	0.478	

* *p*-value < 0.05. SD, standard deviation.

**Table 5:** Multiple linear regression model of factors associated with cognitive attitude and the intention to share misinformation on T2DM.

Cognitive Attitude			Intention to Share		
Factor	Beta (95% CI)	*p*-Value	Factor	Beta (95% CI)	*p*-Value
Educational level: Primary	−1.72 (8.42, 4.99)	0.61	Monthly income (THB) ≤10,000 vs. >10,000	0.67 (−0.22, 1.56)	0.14
Educational level: Above primary	−4.84 (−11.51, 1.83)	0.15	Comorbidity ^#^: present vs. absent	−2.04 (−3.88, −0.20)	0.03 *
Source from the healthcare provider’s score	0.97 (0.43, 1.51)	<0.001 *	Current treatment: oral anti-diabetic drugs	2.01 (0.50, 3.52)	0.01 *
Source from the diabetes patient score	0.48 (0.09, 0.87)	0.02 *	Current treatment: insulin therapy	−0.28 (−2.56, 1.99)	0.80
Model; Adjusted R-squared: 0.20, Residual standard error: 4.64			Source from healthcare provider score	0.40 (0.11, 0.68)	0.01 *
			Attitude score	0.24 (0.15, 0.33)	<0.001 *
			Exposure score	0.46 (0.29, 0.63)	<0.001 *

Model; Adjusted R-squared: 0.50; Residual standard error: 2.20. * *p*-value < 0.05. **^#^** Comorbidity = atrial fibrillation or benign prostate hyperplasia or gout. CI, confidence interval.

## Data Availability

The data that support the findings of this study are available from the corresponding author, Phoomjai Sornsenee, upon reasonable request.

## Abbreviations

The following abbreviations are used in this manuscript:

CI	Confidence interval
T2DM	Type 2 diabetes mellitus
